# Entropy Bathtub for Living Systems: A Markovian Perspective

**DOI:** 10.3390/e28020139

**Published:** 2026-01-25

**Authors:** Krzysztof W. Fornalski

**Affiliations:** Physics of Complex Systems Division, Faculty of Physics, Warsaw University of Technology, ul. Koszykowa 75, 00-632 Warszawa, Poland; krzysztof.fornalski@pw.edu.pl

**Keywords:** physics of life, Markov, entropy, living systems, stochastic thermodynamics, non-equilibrium steady state, bathtub

## Abstract

A living organism can be regarded as a dissipative, self-organizing physical system operating far from thermodynamic equilibrium. Such systems can be effectively described within the framework of Markov jump processes subjected to an external driving force that sustains the system away from equilibrium—leading, in the special case of stabilization, to a non-equilibrium steady state (NESS). By combining the Markov formalism with concepts from stochastic thermodynamics, we demonstrate the temporal evolution of entropy in such systems: entropy decreases during growth and development, stabilizes at maturity under NESS conditions, and subsequently increases during aging, death, and decomposition. This characteristic trajectory, which we term the *entropy bathtub*, highlights the universal thermodynamic structure of living systems. We further show that the system exhibits continuous yet time-dependent positive entropy production, in accordance with fundamental thermodynamic principles. Perturbations of the driving force—whether reversible or irreversible—naturally capture the impact of external stressors, providing a conceptual analogy to pathological processes in biological organisms. Although the model does not introduce fundamentally new elements to the physics of life, it offers a simple tool for exploring entropy-driven mechanisms in living matter.

## 1. Introduction

Let us imagine we are standing on a vast beach, surrounded by a large, flat expanse of sand. The grains of sand move stochastically, and the system is subject to numerous fluctuations. If we take a stick and draw the shape of a living organism—for example, a dog—in the sand, the newly defined system will be in thermodynamic equilibrium with its surroundings. Obviously, sand arranged in the shape of a dog does not mean we are dealing with a living dog. On the contrary: such a system has none of the characteristic features of a living system.

If, however, we arrive at the sandy beach with our actual pet, the dog—as a thermodynamic system—does exhibit the hallmarks of a living system: it is far from thermodynamic equilibrium with the environment, maintains a lower local entropy than its surroundings at an essentially constant level, dissipates energy (into a less organized form) obtained from the environment, and is characterized by specific energy flows—concepts described in detail in the works of Onsager [1] and Prigogine [2,3]. Moreover, as explained by England [4,5], it also possesses the capacity for replication, at least at the cellular level.

To better understand this intriguing process, let us turn to an analogy involving a bathtub filled with water. The humble bathtub has played its part in physics before—most famously in Archimedes’ discoveries. If we fill the bathtub with water and leave it for a sufficiently long time, the bathtub as a system will reach thermodynamic equilibrium with its surroundings (neglecting certain subtleties such as water evaporation or the more complex molecular structure of the bathtub itself as a container). However, if we open the drain at the bottom, the water will begin to flow out, disturbing the system and spontaneously creating a self-organizing structure in the form of a vortex. This results in a local decrease in entropy and drives the system out of thermodynamic equilibrium [6].

Of course, this process will cease as soon as all the water has drained from the bathtub. Therefore, to maintain such a state for a sufficiently long time, we must pump the discharged water back into the bathtub. This can be done using a pump or even by carrying buckets of water—in any case, we must perform work on the system to keep it far from equilibrium and to sustain it at a lower level of entropy (at least in the organizational sense). In effect, we maintain the system in what is known as a steady state, in which the inflow of water equals its outflow—in other words, the thermodynamic flows $J_{i}$ driven by appropriate thermodynamic forces $X_{i}$ are kept at a constant level, and produce entropy, ${\dot{S}}_{int}=\sum_{i}J_{i}X_{i}$.

But how does this relate to our dog? Just like any living animal, a dog must breathe, drink, eat—and consequently excrete and defecate. From time to time, it must also reproduce in order, as in England’s theory of life [4,5], to extend the species by continuously reducing the system’s entropy through replication. We can therefore see a significant analogy between a bathtub with continuously flowing water and a living organism. This does not, of course, mean that a bathtub is alive—but it does share certain features with a living system.

## 2. Entropy Bathtub

**Figure 1:**
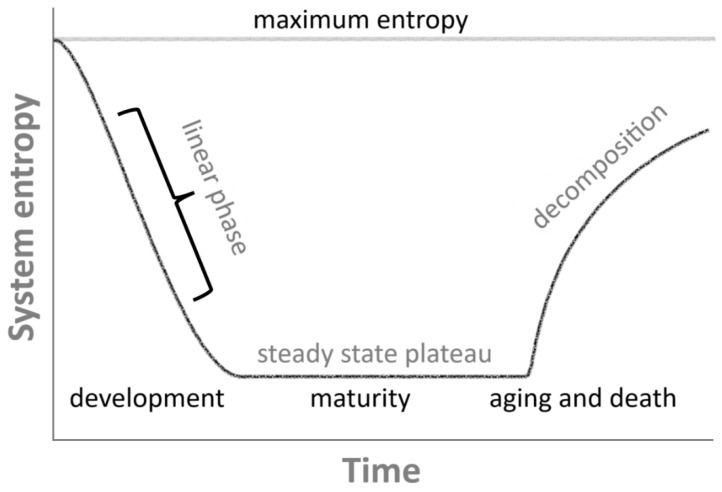
Schematic illustration of the entropy bathtub concept for a living system far from thermodynamic equilibrium: in the initial phase of development (from the moment of fertilization until the organism reaches full maturity), the entropy of the system decreases. In the middle phase, the entropy level stabilizes as the system attains a steady state—corresponding to a constant entropy value over time. As time progresses, the steady state eventually breaks down due to aging processes and, ultimately, death, where the system’s entropy rapidly increases until it slowly approaches thermodynamic equilibrium with the environment (absence of a driving force).

The subject of this conceptual article is the entropy bathtub—a specific, characteristic shape of the time dependence of a system’s entropy ($S_{sys}(t)$), whose profile resembles the cross-sectional shape of a bathtub (Figure 1). This temporal pattern was first described—albeit in a different context—in the textbook of Jaroszyk [7]. It is characterized by an initial phase, corresponding to the early stages of life (from the moment of fertilization), in which the entropy of the system decreases rapidly due to strong self-organization and intensive cell replication (${\dot{S}}_{sys}<0$). Naturally, this also implies a substantial production of entropy (${\dot{S}}_{int}>0$); however, it is balanced by the Prigoginian [2,3] concept of entropy export from the system (${\dot{S}}_{exp}<0$) to the environment for metabolic reasons, resulting in an overall decrease in the system’s entropy (${\dot{S}}_{sys}={\dot{S}}_{int}+{\dot{S}}_{exp}<0$).

Once a living organism reaches adulthood and ceases its intensive growth (e.g., when an animal has attained its final size), the entropy balance levels out, as the system approaches its characteristic steady state (${\dot{S}}_{sys}=0$). The system’s entropy ceases to decrease, yet remains lower than that of the environment. At this stage, the organism is said to have reached the bottom of its entropy bathtub: we called that a biological homeostasis [8,9]. Of course, the entropy profile is not perfectly flat, as the system is subject to numerous fluctuations [10] and sometimes substantial transient deviations from the steady state, for instance due to disease or systemic disruptions. In such cases, the entropy value may temporarily increase but will usually return to its original steady state if the perturbation is not too severe (e.g., recovery from a viral infection).

If, however, the perturbation is significant and irreversible, the entropy may permanently increase, leading to a new steady state—now at a higher entropy level than the original one (new homeostasis [9]). This may occur in situations where the living system undergoes lasting change, such as a permanent injury, a myocardial infarction, or an irreversible stroke. Over time, every living system will experience multiple local and global increases in entropy, reflecting the gradual degradation of the system—in other words, the organism ages. Eventually, death occurs, accompanied by a rapid increase in entropy (${\dot{S}}_{sys}>0$), marking the second edge of the entropy bathtub.

It is worth noting that, while the entropy of the organism indeed rises sharply at the moment of death, it does not reach its maximum (full thermodynamic equilibrium with the environment) immediately. The decomposition of a dead body is a physically, biologically, and chemically complex process, and complete disintegration and chemical equilibration with the surroundings can take a very long time. This is why we are able to find the remains of living organisms even millions of years after their death. The process of decomposition is therefore often much longer than the time required for the system to reach its entropy minimum (from fertilization to adulthood), although this is not always the case.

## 3. Markov Jump Processes

There exist numerous theoretical models describing the development of a living organism in the context of its entropy. From the perspective of statistical physics and stochastic thermodynamics, a particularly useful—though necessarily simplified—theoretical analogue of a living system is a stochastic Markovian system [11,12,13]. Its advantage lies in the ability to represent the system at multiple energy levels, in various states, each described by its own probability distribution. Such a stochastic framework captures, at a certain level of generality, the complexity of a living system—while remaining sufficiently accurate to reflect its essential components and processes.

It should be emphasized that the Markov jump framework employed here is not intended as a microscopic description of biological dynamics. Rather, it constitutes an effective, coarse-grained representation, in which the complex nonlinear interactions between biochemical, mechanical, and informational subsystems are implicitly encoded in the transition rates [14,15]. At this mesoscopic level, stochasticity does not imply randomness in the biological sense, but reflects the aggregation of many underlying deterministic and nonlinear processes into probabilistic transitions between states. Such an approach is standard in statistical physics and stochastic thermodynamics, where Markovian dynamics successfully capture irreversible behavior despite the underlying nonlinearity of the full system [16].

**Figure 2:**
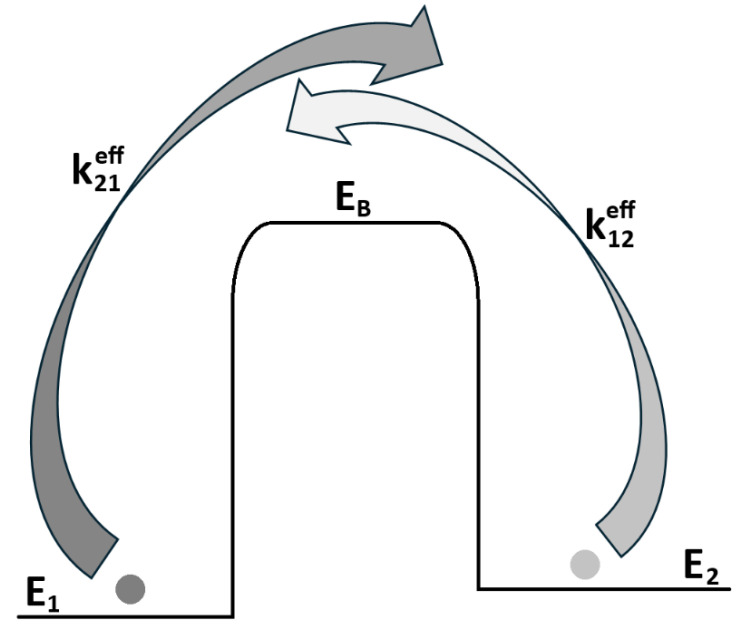
The two-state Markovian jump system: an exemplary case in which a particle jumps between two states with energies $E_{1}$ and $E_{2}$, separated by a potential barrier $E_{B}$. Both effective jump rates, $k_{12}^{eff}$ and $k_{21}^{eff}$, describe transitions between the two states without explicitly including the barrier state.

Naturally, many types of Markovian systems exist, ranging from simple to highly complex. To illustrate their relevance for simulating a living system and for determining the entropy bathtub profile, let us begin with the simplest case: the classical two-state jump Markovian system. In such a system—schematically depicted in Figure 2—we consider two energy states, denoted $E_{1}$ and $E_{2}$, occupied by particles with probabilities $p_{1}$ and $p_{2}$, respectively. The states are separated by a fixed potential barrier $E_{B}$, which is implicitly accounted for in the transition rates k (here we adopt the convention that a transition from state $E_{1}$ to $E_{2}$ is denoted as $k_{21}$).

The corresponding Master equation for the situation shown in Figure 2 is:

(1) $$\left\{\begin{array}{c} {\dot{p}}_{1}=k_{12}^{eff}\;p_{2}-k_{21}^{eff}\;p_{1}\\ {\dot{p}}_{2}=k_{21}^{eff}\;p_{1}-k_{12}^{eff}\;p_{2} \end{array}\right.$$

where $p_{x}$ is a probability distribution of given state *x*, and $k_{xx^{\prime }}^{eff}$ is an effective jump rate between two states (its effectivity means that the jump omits the state located on the top of potential barrier). In the situation of equilibrium, both jump rates satisfy the detailed balance (will be discussed later). Assuming a common intrinsic rate of jumps as *ω*, one can get

(2) $$\left\{\begin{array}{c} k_{21}^{eff}=\frac{\omega }{2}e^{\beta \left(E_{1}-E_{B}\right)}\\ k_{12}^{eff}=\frac{\omega }{2}e^{\beta \left(E_{2}-E_{B}\right)} \end{array}\right.$$

which corresponds to the Arrhenius equation, with activation energy barriers $E_{B}-E_{1}$ and $E_{B}-E_{2}$, respectively. Here $k_{xx^{\prime }}^{eff}$ are, to first approximation, time-independent.

Of course this two-state system, as presented above, has its equilibrium condition written in the form of detailed balance as:

(3) $$\frac{k_{21}^{eff}}{k_{12}^{eff}}=e^{\beta \left(E_{1}-E_{2}\right)}=\frac{p_{2}^{eq}}{p_{1}^{eq}}$$

In general, however, the system may be subjected to an external directional driving force $\delta_{xx^{\prime }}$, which can represent work done on the system (as described in the Introduction). Under such forcing, one of the jump rates may be significantly favoured, leading to a departure from equilibrium. This is the fundamental mechanism for sustaining nonequilibrium states via an external thermodynamic drive.

**Figure 3:**
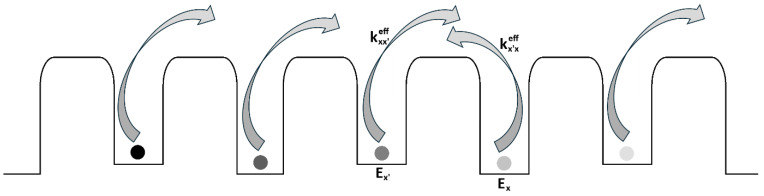
The Markovian jump system for multiple states: particles moving along a chain of potential wells ($E_{x}$) separated by barriers ($E_{B}$), with effective jump rates $k^{eff}$ between them. This structure can serve as an analogy to a DNA chain [17].

The simple two-state model serves as an academic starting point for understanding the statistical physics of Markovian systems. For a living system, however, it is an oversimplification. We can extend the model by introducing many (though not infinitely many) potential wells separated by barriers—thus generalizing to a multi-state Markovian jump process, schematically illustrated in Figure 3.

**Figure 4:**
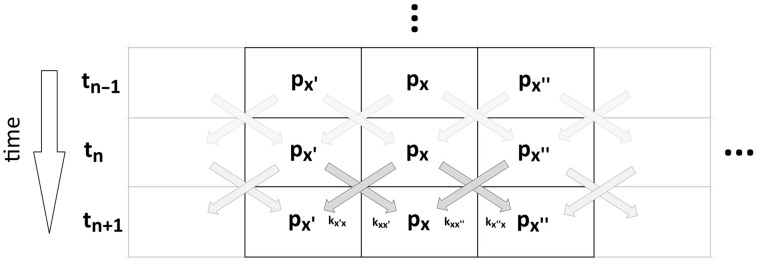
The scheme from Figure 3 is presented in the form of a simplified numerical algorithm, where each horizontal line represents the arrangement of states in a Markov chain at a given moment in time (analogous to Figure 3), while the vertical downward axis schematically denotes the (discrete) time axis. The diagonal arrows represent transitions between states with the corresponding jump rates, *k*. Ellipses indicate that the array can be arbitrarily long throughout the entire numerical simulation, both in terms of the number of states and the number of time steps.

The same process, but decomposed step by step for each moment in time, is schematically illustrated in Figure 4. This scheme can be readily applied as a numerical algorithm.

In that situation, let us formulate more general Master equation as

(4) $${\dot{p}}_{x}=\sum_{x^{\prime }(\neq x)}\left(k_{xx^{\prime }}^{eff}\;p_{x^{\prime }}-k_{x^{\prime }x}^{eff}\;p_{x}\right)$$

The right-hand term in brackets can be simply replaced by probability current (flow) from the state x′ to x, denoted as $J_{xx^{\prime }}$, therefore ${\dot{p}}_{x}=\sum J_{xx^{\prime }}$ which is a non-zero value for mutating and evolving system, e.g., living thing with DNA chain. In that case nonvanishing current can only be sustained if the rates pushing the currents in one direction do not balance [16,18].

Analogically, the effective jump rate for the multi-stage system from Figure 3 can be presented as $k_{xx^{\prime }}^{eff}=\frac{1}{2}\omega \;\exp\left[\beta \left(E_{x^{\prime }}-E_{B}+\delta_{xx^{\prime }}\right)\right]$ with the one-sided driving force $\delta_{xx^{\prime }}>\delta_{x^{\prime }x}\geq 0$. Please note, that for the particle jump from *x′* to *x*, the heat released from the system into the environment (reservoir) equals $Q_{xx^{\prime }}=E_{x^{\prime }}-E_{x}+\delta_{xx^{\prime }}$ where $\delta_{xx^{\prime }}$ is the mentioned energy provided by external driving (external force facilitating the jump), for example the work done on the system to keep its steady state (see the analogy to the water pump in water bathtub). Interpreting Figure 3 as an analogy for a DNA chain [17], one can associate the driving force with an external evolutionary stressor such as ionizing radiation, which deposits energy per unit mass and serves as a potent driver of DNA mutation and evolution. In the simplest case of spontaneous mutation, this factor is zero: $\delta_{xx^{\prime }}=0$.

The presented multi-state Markovian process is already a suitable tool for simulating far-from-equilibrium living systems. Figure 3 shows the simplest linear arrangement, which can be extended to higher dimensions. This increases computational calculations complexity but also improves the model’s ability to represent the intricacy of the systems under study.

Therefore, let us define the most general multidimensional continuous-time Markovian jump process given by its matrix Master equation:

(5) $$\dot{p}=K\cdot p$$

where p is the probability vector with components $p_{x}$ (where $\sum_{x}p_{x}=1$), and K is the transition rate matrix—its off-diagonal elements are $K_{xx^{\prime }}=k_{xx^{\prime }}^{eff}$, and diagonal elements are $K_{x^{\prime }x^{\prime }}=-\sum_{x^{\prime }\neq x}k_{x^{\prime }x}^{eff}$. Each jump rate (transition rate) can be therefore generalized as $k_{xx^{\prime }}^{eff}\sim \exp\left[B\pm S/2\right]$, where B and S parameterize the symmetric and antisymmetric component of the transition rate, respectively. The symmetric term B is responsible for potential barriers while antisymmetric S is responsible for the entropy change associated with transitions that involve modifications of thermodynamic driving forces and the energy landscape [10].

## 4. Entropy in Markovian Systems

Let us focus on the entropy during the jump between two states: applying classical Prigoginian formula for the total entropy [2] one obtains $S_{tot}=S_{sys}+S_{res}$ where $S_{sys}$ represents the internal system entropy, and $S_{res}$ denotes the entropy of reservoir (thermal bath) around the system.

In the simplest approach, the system entropy can be calculated using the classical definition of Shannon entropy, which results in

(6) $$S_{sys}=-k_{B}\sum_{x}p_{x}\ln p_{x}$$

which is true for the dedicated system configuration in a particular moment of time. Consequently, the system entropy change, $∆S_{sys}$, for example between two consecutive Markovian jump processes, is the difference between the entropy obtained by Equation (6).

Alternatively, one may employ the Schnakenberg framework for non-equilibrium Markovian systems [19] to calculate the system entropy change:

(7) $$∆S_{sys}=\frac{1}{2}k_{B}\;∆t_{jump}\sum_{x^{\prime }(\neq x)}J_{xx^{\prime }}\;\ln\frac{\;p_{x^{\prime }}}{\;p_{x}}$$

with the probability current $J_{xx^{\prime }}$ based on the Master equation, see Equation (4). Please note that ½ prevents the double counting of the current between every pairs of *x*s.

In essence, there is no major difference whether we evaluate entropy dynamics using Equation (7) or via entropy differences from Equation (6). Both approaches yield qualitatively similar results. However, for states far from equilibrium, Equation (7) often better captures certain fluctuation-related behaviors. Anyway, in the calculations carried out in this article, the entropy was ultimately taken as that computed using the classical Shannon formula given by Equation (6) [15].

The calculation of entropy change is crucial for demonstrating the entropy decrease in a system during its developmental phase—i.e., before the adulthood and stabilization period described in previous sections (which we defined as the non-equilibrium steady state, NESS). In this stage, the system exhibits a continuous decrease in entropy ($∆S_{sys}<0$), often approximately linear in time ($∆S_{sys}=const<0$) [20,21], see Figure 1. After a sufficiently long period (analogous to the relaxation time under constant driving force), the system stabilizes upon reaching maturity, and the probability currents vanish ($\sum J_{xx^{\prime }}=0$). The system entropy then settles at its lowest attainable value, and the entropy change is zero:

(8) $$\sum_{x^{\prime }(\neq x)}\left[p_{x^{\prime }}^{st}\;e^{\beta \left(E_{x^{\prime }}+\delta_{xx^{\prime }}\right)}-p_{x}^{st}\;e^{\beta E_{x}}\right]=0\;\;\;$$

with $p_{x^{\prime }}/p_{x}=const\equiv \;p_{x^{\prime }}^{st}/p_{x}^{st}$. At this point, the system has reached the “bottom” of the entropy bathtub, which will vanish once the driving force ceases and the system relaxes back to thermodynamic equilibrium. Parallelly one has to note that the stationary distribution of *p_x_* (which is a probability distribution over mesostates) can be a non-equilibrium steady state (NESS) under two formal conditions [16]:

(9) $$\left\{\begin{array}{c} \sum_{x^{\prime }(\neq x)}J_{xx^{\prime }}=0\\ k_{x_{0}\;x_{1}}^{eff}\;k_{x_{1}\;x_{2}}^{eff}\ldots \;k_{x_{n}\;x_{0}}^{eff}\neq k_{x_{0}\;x_{n}}^{eff}\;k_{x_{n}\;x_{n-1}}^{eff}\ldots \;k_{x_{1}\;x_{0}}^{eff} \end{array}\right.$$

which is the case in our situation.

**Figure 5:**
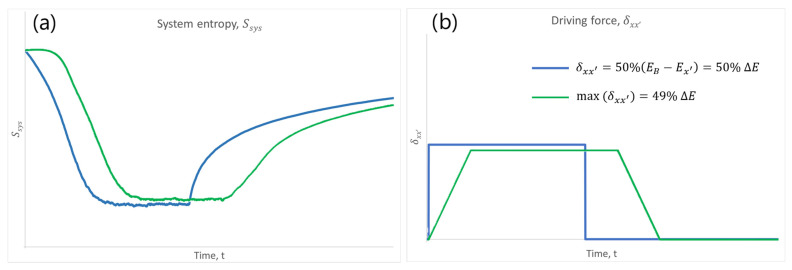
Representative qualitative results of numerical simulations for the system presented in the schematic of Figure 4. The Markov system, composed of 30 potential wells and initially at thermodynamic equilibrium, was subjected to a unidirectional driving force that drove it into a non-equilibrium state, producing the characteristic “entropy bathtub” (panel (**a**)). The driving force (panel (**b**)) had either a rectangular time profile (blue curve) or a trapezoidal profile (green curve). Initial jump rates (at zero driving force) were randomly drawn from a uniform distribution in the range [0.01, 0.02]; initial probability distributions were randomly drawn from a uniform distribution; and all other fixed parameters were set to unity.

Figure 5 illustrates two exemplary entropy bathtubs: in the first case (blue curve), the driving force appears suddenly, remains constant for some time, and then drops back to zero (Figure 5a). In the second case (green curve), the driving force increases linearly over time, reaches a plateau, and then decreases linearly back to zero (Figure 5b). In both cases, a characteristic entropy bathtub profile emerges, although its detailed shape depends on the temporal form of the driving force.

The numerical results presented in Figure 5 were obtained by simulating a continuous-time Markovian jump process defined on a one-dimensional chain of 30 potential wells, as schematically illustrated in Figure 3. The simulations were implemented using a ROOT numerical code based on direct calculation of the Master equation with discrete time steps. Unless stated otherwise, all results correspond to single representative realizations, as the qualitative behavior of the entropy bathtub was found to be robust with respect to random initial conditions and moderate variations in model parameters.

The initial probability distributions were drawn from a uniform distribution, while the initial jump rates (at zero driving force) were sampled uniformly from the interval [0.01, 0.02]. Time in the simulations is expressed in dimensionless units set by the inverse intrinsic jump rate, and therefore represents a rescaled physical time. The relaxation time of the system, defined as the characteristic time required to reach a non-equilibrium steady state under constant driving, is of the same order in all simulations shown in Figure 5. Consequently, the time axes in Figure 5a,b correspond to comparable dynamical regimes, despite the different temporal profiles of the driving force. The specific rectangular and trapezoidal driving-force protocols were chosen for illustrative purposes, as minimal examples demonstrating how the qualitative shape of the entropy bathtub depends on the temporal structure of the external drive. The goal of these simulations is not quantitative prediction, but rather the demonstration of generic entropy-driven behavior in a minimal stochastic model.

## 5. Discussion

This article presents the concept of the “entropy bathtub” for systems far from thermodynamic equilibrium (e.g., living systems), understood as the time evolution of entropy in such a system. The “entropy bathtub” was simulated using a classical Markovian system with potential wells and barriers, together with the corresponding jump rates. The concept itself is not entirely new [7], as it reflects well-known aspects of non-equilibrium statistical physics. Nevertheless, its novelty lies in providing a unified, general description of the behavior of living systems, as well as in its explicit numerical implementation within the framework of Markov processes.

Many recent theoretical and experimental studies have further refined the role of entropy-related measures in biological development by introducing concepts such as patterning entropy and reproducibility entropy. In particular, Brückner and Tkačik [22] demonstrated that precise and reproducible developmental programs correspond to entropy minimization under biological constraints. Complementary experimental evidence from multicellular systems, such as early embryonic patterning in Drosophila [23] and spinal cord development [24], shows that living systems actively reduce configurational uncertainty during development in order to achieve functional organization.

These results are fully consistent with the entropy bathtub framework proposed here. The initial decreasing branch of the entropy bathtub corresponds to a phase of entropy minimization driven by developmental constraints and selection of reproducible patterns, while the subsequent non-equilibrium steady state reflects the maintenance of such patterns against fluctuations through continuous entropy production and export.

**Figure 6:**
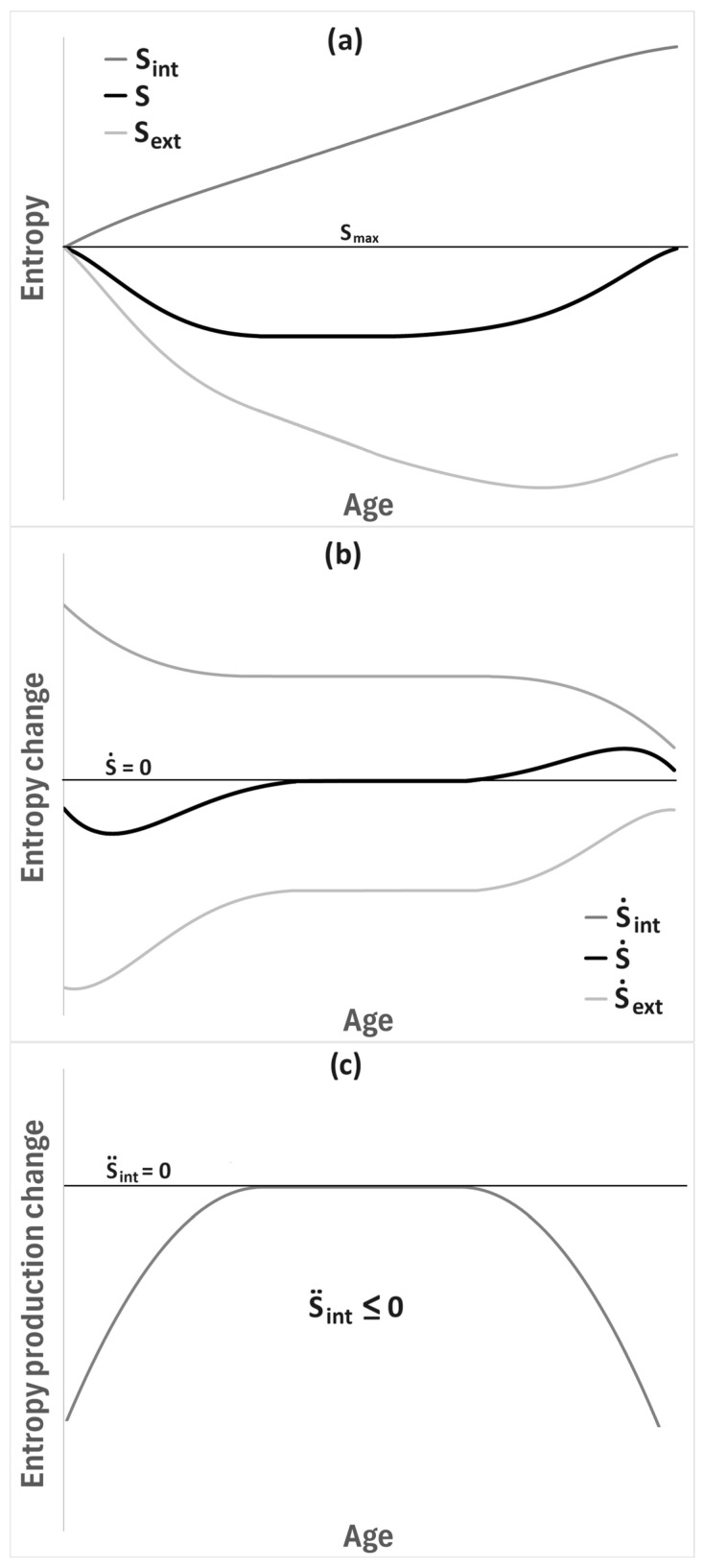
The exemplary possible qualitative shape of entropy, entropy change and its components in the function of time (age) for the living organism. Plot (**a**) shows how the value of entropy is dependent on time: middle black line, which has a “bathtub” shape, represents the organism’s internal entropy together with its components related to the entropy already produced with time (upper grey curve) and entropy fluxed (lower grey curve). Plot (**b**) shows entropy change: overall entropy change (black middle curve), entropy production (${\dot{S}}_{int}$) and entropy flux (${\dot{S}}_{ext}$); one can observe when the young organism produce more entropy ($\dot{S}<0$), when stationary state is reached ($\dot{S}=0$), and when old organism loses its entropy ($\dot{S}>0$); please note that the entropy production curve (upper one) has purely experimental background [25]. Plot (**c**) represents the entropy production change, which fulfills Glansdorff–Prigogine criterion of system stability, ${\ddot{S}}_{int}\leq 0$ [26].

Let us now examine the entropy bathtub from a purely thermodynamic perspective. Figure 6a illustrates the time evolution (interpreted as a hypothetical age of the living system) of system entropy under conditions of departure from thermodynamic equilibrium. The system entropy forms the previously described bathtub-like profile (bold black curve), which never exceeds the maximum entropy level indicated by the horizontal line. Attention should also be paid to the two additional curves (thin gray lines): the monotonically increasing internal entropy of the system, consistent with the Second Law of Thermodynamics, and the decreasing entropy exchanged with the environment as a consequence of metabolic processes. Thus, the total entropy of the system is the sum of these two contributions, in accordance with the framework of Prigogine [2,3].

To better characterize the evolution of these quantities, their time derivatives are schematically presented in Figure 6b. In the steady state, the time derivative of system entropy vanishes, corresponding to the “bottom” of the entropy bathtub and to the constant reduced entropy level (Figure 6a). Accordingly, for a developing system one finds $-{\dot{S}}_{exp}>{\dot{S}}_{int}>0$; for a system in the steady state, ${\dot{S}}_{int}+{\dot{S}}_{exp}=0$ (which does not necessarily imply that both terms are individually constant in time—although such the simplest case is depicted in Figure 6b); and for a degrading system, ${\dot{S}}_{int}>-{\dot{S}}_{exp}>0$. Importantly, examining the upper curve in Figure 6b reveals that, regardless of the developmental stage, entropy is continuously produced within the system (${\dot{S}}_{int}>0$), which is fully consistent with the general reasoning for systems far from equilibrium. Moreover, this profile agrees with recent experimental observations [25].

Of particular interest is Figure 6c, which shows the second time derivative of the internal entropy, i.e., the entropy production rate. One observes that, irrespective of the developmental stage, ${\ddot{S}}_{int}\leq 0$. This corresponds to the Glansdorff–Prigogine criterion [26], which characterizes the stability of systems out of thermodynamic equilibrium. More precisely, the criterion states that for a system maintained in a non-equilibrium steady state, the excess entropy production must remain non-negative, ensuring that small fluctuations or perturbations cannot spontaneously destabilize the system. In the context of the present work, the fact that ${\ddot{S}}_{int}\leq 0$ at all times implies that the entropy production monotonically relaxes as the system evolves, and thus the entropy bathtub remains dynamically stable throughout development, steady-state maintenance, and eventual degradation. (As can be seen, this criterion is satisfied in every phase of the entropy bathtub, although the Glansdorff–Prigogine condition was originally formulated for processes close to thermodynamic equilibrium, where the Onsager relations hold [1].) This reinforces the interpretation that the entropy bathtub is not merely a phenomenological picture, but a thermodynamically consistent description of living systems as dissipative structures operating under continuous entropy export to their surroundings.

Please note that the presented model does not explicitly impose a variational principle for entropy production. Nevertheless, its behavior is fully consistent with the established thermodynamic principles governing non-equilibrium systems. In regimes close to equilibrium, the dynamics of dissipative structures are known to obey Prigogine’s principle of minimum entropy production. By contrast, biological systems typically operate in steady states far from equilibrium, where their evolution is often discussed in the context of the maximum entropy production principle (MEPP) [27,28]. In the Markovian framework adopted here, the system is driven by external forces that sustain non-vanishing probability currents and continuous entropy production. While the transition rates are prescribed rather than optimized, the resulting steady states exhibit sustained entropy export and stable internal organization, in qualitative agreement with the notion that far-from-equilibrium biological systems evolve along paths compatible with maximal dissipation under given constraints.

Finally, it should be emphasized that there is no universal rule prescribing the sign of entropy change when a system is driven out of equilibrium. Depending on initial conditions, constraints, and the nature of the driving, the internal entropy of a system may either increase or decrease relative to its initial value. In the context of living systems, the decreasing branch of the entropy bathtub corresponds to a biologically relevant subset of non-equilibrium trajectories associated with self-organization and functional development, rather than a general property of driven systems.

At this point, it is instructive to note that the bathtub-shaped profile of entropy discussed here bears a close conceptual resemblance to the well-known *bathtub curve* from reliability engineering and failure theory [29,30,31]. In that context, the bathtub curve describes the time dependence of failure rates in technical systems, comprising three characteristic phases: an initial period of high failure probability (“infant mortality”), a long interval of approximately constant and minimal failure rate (“useful life”), and a final phase of rapidly increasing failure probability associated with wear-out and material degradation.

Although traditionally formulated for non-living, engineered systems, the reliability bathtub curve reflects a much more general principle that transcends the distinction between the living and the non-living. In technical systems, early failures are typically associated with latent defects, imperfect assembly, or insufficient stabilization, whereas late failures arise from the cumulative effects of irreversible damage, fatigue, and entropy accumulation. In living systems, these stages find their natural counterparts in early developmental fragility, mid-life homeostatic stability, and late-life degenerative decline [32].

From a thermodynamic perspective, the entropy bathtub introduced in the present work may thus be viewed as a fundamental counterpart of the reliability *bathtub curve*, formulated not in terms of failure rates but in terms of entropy balance and entropy production in systems maintained far from equilibrium. In both cases, the central “flat” region corresponds to a dynamically stable regime: in reliability theory, this is characterized by approximately constant hazard rates, whereas in the entropy bathtub it corresponds to a non-equilibrium steady state with vanishing total entropy change and sustained entropy export to the environment.

Importantly, the analogy also highlights a crucial difference. While technical systems passively accumulate damage until failure becomes inevitable, living systems actively counteract entropy accumulation through metabolism, repair, and adaptation, thereby stabilizing the bottom of the entropy bathtub over extended periods. Nevertheless, in both living and non-living systems, the eventual loss of this balance in NESS leads to an irreversible transition toward equilibrium, manifested either as system failure or biological aging and death. In this sense, the entropy bathtub provides a unifying thermodynamic framework that naturally bridges reliability theory, non-equilibrium physics, and the life-cycle dynamics of complex systems.

It is important to stress that the increase in entropy discussed here should not be interpreted as a simple loss of structure in the sense of closed-system thermodynamics. Living organisms are open systems that continuously exchange matter, energy, and entropy with their environment. Local decreases or stabilization of internal entropy are therefore fully compatible with the Second Law, provided that sufficient entropy is exported to the surroundings. Aging, within the present framework, is not described as a monotonic randomization of microscopic configurations, but rather as a gradual degradation of the system’s ability to sustain non-equilibrium steady states under fixed environmental constraints [33].

A particularly illustrative and non-trivial case in this context is provided by cancer [34]. From the perspective of the entropy bathtub and the associated bathtub-curve analogy, a tumor may be viewed as a subsystem that effectively circumvents the final wear-out (equilibration) phase of the bathtub curve. While the host organism progressively exits the non-equilibrium steady state and enters the degradation regime, the tumor locally maintains a state resembling persistent NESS, characterized by sustained proliferation, high metabolic activity, and continuous entropy export. In thermodynamic terms, this corresponds to a spatial and functional decoupling of entropy balance: the tumor stabilizes its own local entropy bathtub at the expense of increasing entropy production and structural degradation in the surrounding tissue and the organism as a whole. Thus, cancer does not violate the entropy bathtub framework but rather exploits it, redistributing entropy production in a way that preserves local non-equilibrium stability while accelerating global system failure [34].

Let us go back to living systems and Figure 6. The bottom of the entropy bathtub is not fixed and immutable throughout the entire lifespan of an organism. In addition to natural stochastic fluctuations, which are clearly visible in Figure 5, the organism is subjected to various types of reversible and irreversible external stimuli (stressors) which—most generally—lead to temporary or permanent changes in the entropy level of the system.

In Markov systems, any stressor acting on the system in a far-from-equilibrium state can be characterized in terms of an opposing driving force, $\delta_{x^{\prime }x}$, which perturbs the original driving force $\delta_{xx^{\prime }}$. Such a representation of the action of a stressor (or stressors) enables a general account of the influence of external factors on the system—whether through altering the heights of potential barriers [17], the depths of potential wells, or any other parameters that modify the jump rates between states. This approach was already discussed in connection with the antisymmetric coefficient *S* of the transition matrix K, which is responsible for entropy changes associated with transitions that generally modify the driving forces and the energy landscape [10].

**Figure 7:**
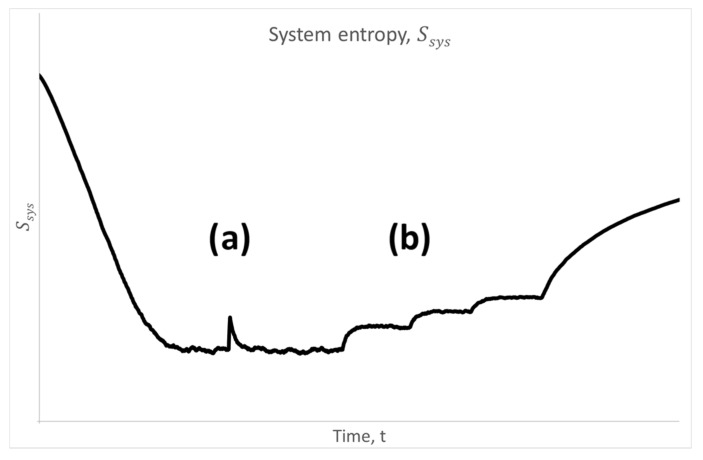
Entropy bathtub identical to Figure 5a, where during the non-equilibrium steady state (the bottom of the bathtub) an external stressor in the form of an opposing driving force was introduced: in case (a) this is a short-term reversible impulse ($∆\delta_{x^{\prime }x}$), whereas in case (b) it is time-constant stressors ($∆\delta_{x^{\prime }x}=const$), irreversibly shifting the system to three exemplary new non-equilibrium steady states (NESSs) with a higher entropy level than the original one. Simulation conditions are the same as in Figure 5.

Let us now consider a short-term and small perturbation in the form of $∆\delta_{x^{\prime }x}$. Such a perturbation induces a local and reversible increase in the system’s entropy, as shown in Figure 7 (case a). Once $∆\delta_{x^{\prime }x}$ vanishes, the system exponentially relaxes back to the original non-equilibrium steady state (NESS). In practice, this may correspond to relatively mild stressors in a living organism, such as viral infections or curable diseases.

The second case involves irreversible diseases (e.g., paralysis, myocardial infarction, or cerebral hemorrhage), after which a constant nonzero opposing driving force persists ($\delta_{x^{\prime }x}=const$). In this scenario (illustrated as case b in Figure 7), the system transitions to a new NESS; however, at a higher entropy level than the original one. This is, of course, associated with degenerative processes (including organismal aging), which—as previously discussed—ultimately lead to exiting the entropy bathtub toward full thermodynamic equilibrium. A constant opposing driving force induced by external stressors can only accelerate this process.

In each of the cases described, as long as the entropy of the system remains lower than the maximum, entropy production takes place, which is a characteristic feature of every living system. This can be expressed by Schnakenberg’s equation [16], through which one can obtain the net average total entropy production rate as:

(10) $${\dot{S}}_{tot}=\frac{1}{2}k_{B}\sum_{x^{\prime }(\neq x)}J_{xx^{\prime }}\;\ln\frac{k_{xx^{\prime }}^{eff}\;p_{x^{\prime }}}{k_{x^{\prime }x}^{eff}\;p_{x}}\;$$

Please note that each term on the right part of Equation (10) is greater than zero (or equals to zero), which means that the total entropy production cannot be negative. However, it goes to zero when $\sum J_{xx^{\prime }}\to 0$ which means that the current between each pair of states vanishes. This situation meets the detailed balance and finally the equilibrium state.

To supplement the information provided above, it is worth mentioning that the continuous form of entropy production function, namely the Schnakenberg formula given by Equation (10), can be expressed as [16]

(11) $$\langle {\dot{S}}_{tot}\rangle =k_{B}\int \frac{J^{2}(x,t)}{D\;p(x,t)}\;dx$$

where *D* is a diffusion constant for probability current. This is the most general case which can be applied to many different systems.

The examples discussed above pertain to living systems, such as individual organisms or cellular colonies. However, one can also envision much more complex systems, such as social networks or cities. Naturally, the analysis of such systems is primarily a matter of scale, yet the physics of complex systems—including econophysics and sociophysics—also offers many intriguing insights. For instance, the processes of growth, stabilization, and decline observed in cities such as Detroit (USA) can likewise be interpreted in terms of entropy evolution, forming a characteristic bathtub-shaped trajectory [35,36,37,38,39]. Nevertheless, such an extension would require dedicated, system-specific investigations.

Of course the model presented in this work is intentionally minimal and conceptual [40]. By construction, it does not resolve the detailed biochemical, genetic, or regulatory mechanisms underlying biological function, nor does it explicitly incorporate nonlinear feedbacks or signaling pathways. Instead, such effects are effectively subsumed into time-dependent transition rates within a coarse-grained Markovian description [14,16]. Consequently, the model should not be interpreted as a predictive biological theory, but rather as a thermodynamically consistent framework for exploring general entropy-driven features of living systems [15,40].

Furthermore, while the analysis explicitly accounts for entropy production and entropy exchange with the environment, it does not attempt to derive the dynamics from a variational extremum principle. The results should therefore be understood as illustrative of generic non-equilibrium behavior rather than as a proof of optimality in the sense of maximum entropy production [14,16,27,28]. Despite these limitations, the simplicity of the framework constitutes its main strength, allowing transparent insight into the interplay between entropy, non-equilibrium steady states, and system degradation.

## 6. Conclusions

This article has demonstrated the application of the formalism of Markovian jump processes, both in a simple two-state configuration and in a more complex multistate system. By employing this framework together with a straightforward numerical model, we have shown that a system driven by a constant unidirectional driving force can successfully simulate the essential features of a living system. Moreover, by perturbing this driving force—either reversibly or irreversibly—we are able to illustrate clear analogies to pathological conditions affecting living organisms.

The Markov model also revealed that a living organism, as a system far from thermodynamic equilibrium, is characterized by continuous entropy production and time-dependent internal entropy. The time profile of entropy assumes a distinctive bathtub-like shape, termed here the entropy bathtub: in the first stage, entropy decreases during growth and development (under suitable biological and environmental constraints); in the second stage, entropy stabilizes at a constant level as the system reaches a non-equilibrium steady state; in the third stage (the second slope of the bathtub), entropy increases due to degradation processes associated with aging, death, and decomposition [41].

The presented framework thus unifies several well-established properties of living systems into a coherent formalism, providing an example of an entropy-driven mechanism. It enables the description of multiple intriguing effects observed in biological systems. Therefore, the model serves as both an insightful conceptual tool and a relatively simple formalism, with promising potential for a wide range of future applications.

## Data Availability

No new data were created or analyzed in this study. Data sharing is not applicable to this article.
